# Supplementary figures and images for: Pak1 kinase controls cell shape through ribonucleoprotein granules
Source: eLife. 2021 Jul 20;10:e67648. doi: 10.7554/eLife.67648 (PMC8318594; doi:10.7554/eLife.67648)

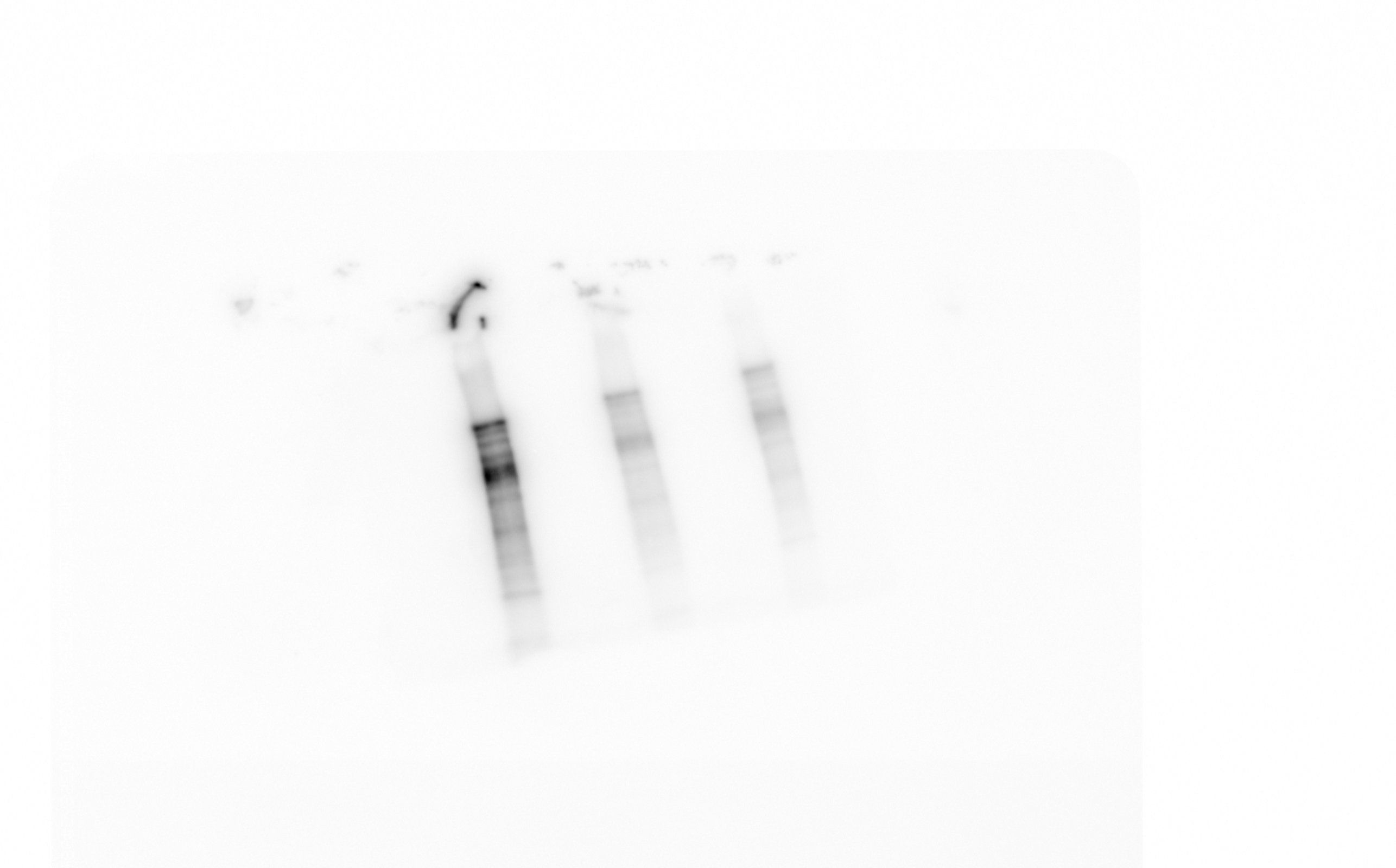

Supplement: Figure 1—source data 2. [file elife-67648-fig1-data2.tif]

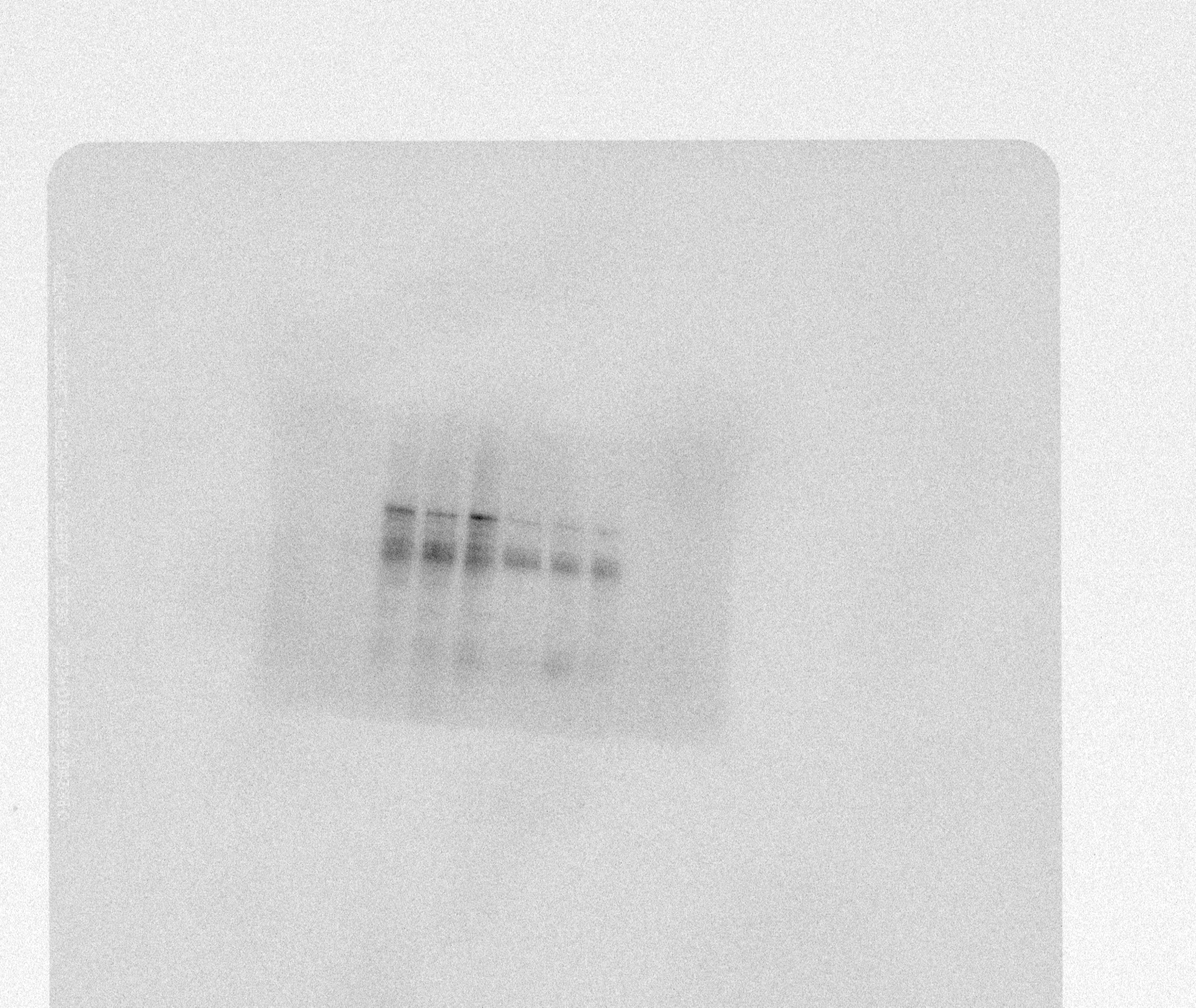

Supplement: Figure 1—source data 3. [file elife-67648-fig1-data3.tif]

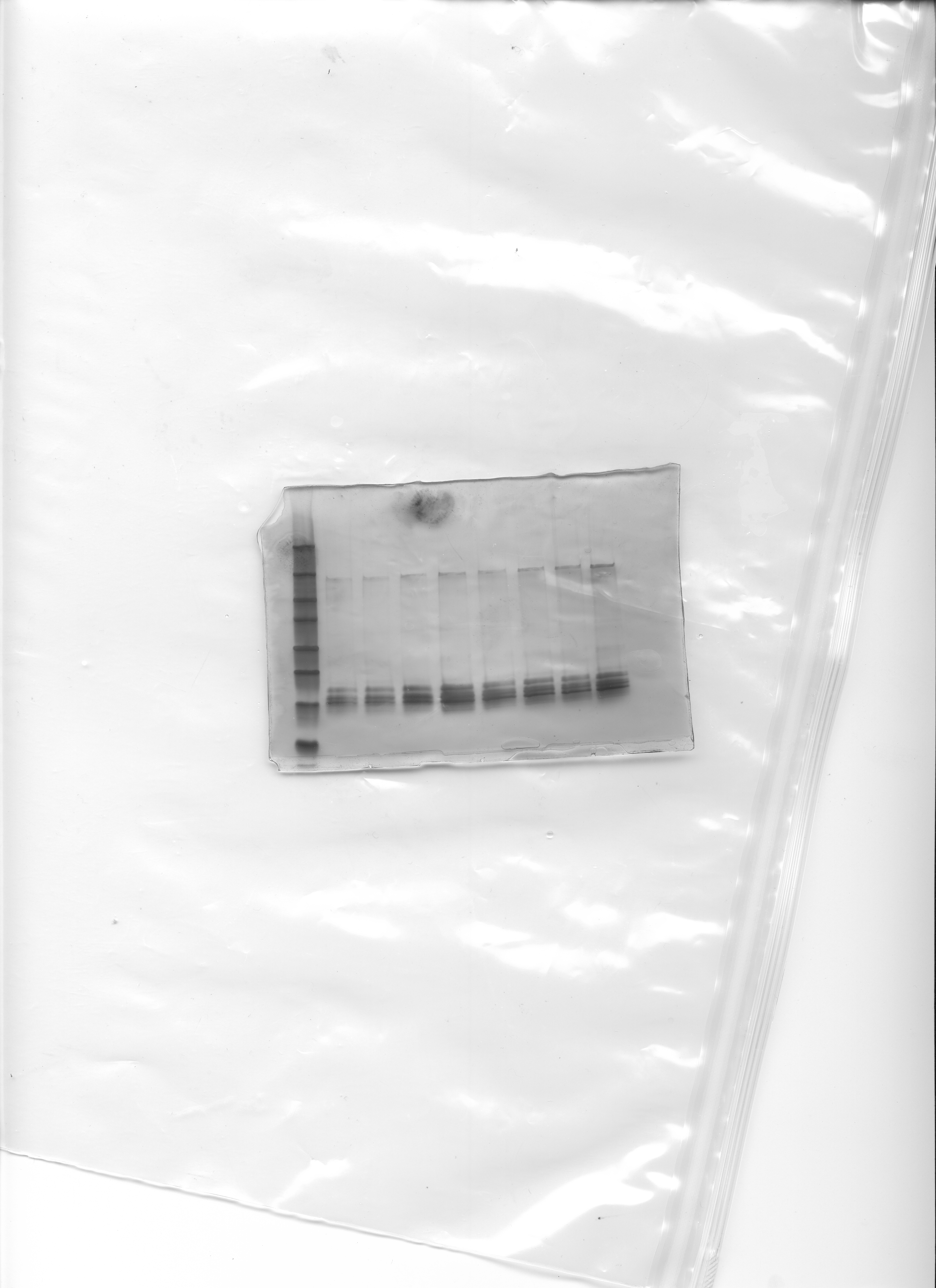

Supplement: Figure 1—source data 4. [file elife-67648-fig1-data4.tif]

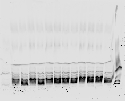

Supplement: Figure 2—source data 2. [file elife-67648-fig2-data2.zip › 0006354_01/0006354_01_TH.jpg]

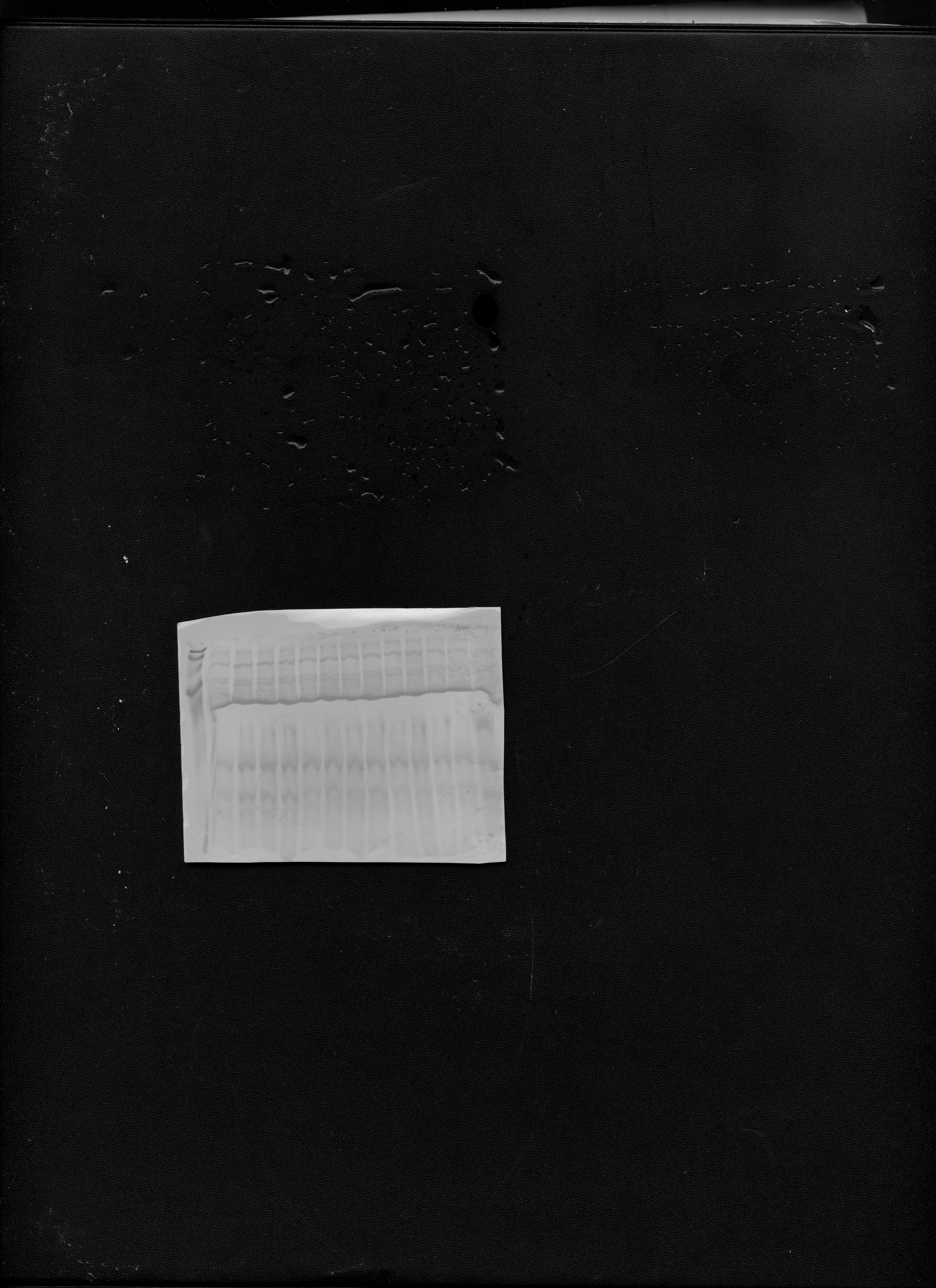

Supplement: Figure 2—source data 3. [file elife-67648-fig2-data3.tif]

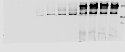

Supplement: Figure 3—source data 2. [file elife-67648-fig3-data2.zip › 0007255_01/0007255_01_TH.jpg]

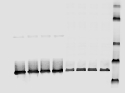

Supplement: Figure 3—source data 3. [file elife-67648-fig3-data3.zip › 0007256_01/0007256_01_TH.jpg]

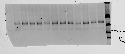

Supplement: Figure 4—source data 2. [file elife-67648-fig4-data2.zip › 0005714_01/0005714_01_TH.jpg]

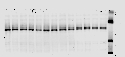

Supplement: Figure 4—source data 3. [file elife-67648-fig4-data3.zip › 0005712_01/0005712_01_TH.jpg]

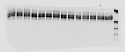

Supplement: Figure 4—source data 4. [file elife-67648-fig4-data4.zip › 0007790_01/0007790_01_TH.jpg]

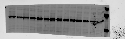

Supplement: Figure 4—source data 5. [file elife-67648-fig4-data5.zip › 0007791_01/0007791_01_TH.jpg]
